# Association between social determinants of health and hearing loss in South African children: A secondary data analysis

**DOI:** 10.1371/journal.pgph.0004790

**Published:** 2025-06-24

**Authors:** Mukovhe Phanguphangu, Andrew John Ross, Tracey Smythe

**Affiliations:** 1 Department of Public Health and Policy, London School of Hygiene and Tropical Medicine, London, United Kingdom; 2 Department of Family Medicine, University of KwaZulu-Natal, Durban, South Africa; 3 International Centre for Evidence in Disability, London School of Hygiene & Tropical Medicine, London, United Kingdom; 4 Division of Physiotherapy, Stellenbosch University, Cape Town, South Africa; PLOS: Public Library of Science, UNITED STATES OF AMERICA

## Abstract

Globally, 34 million children below 15 years have hearing loss (HL) and while research shows associations between social determinants of health and disability in general, research on the associations between these determinants and HL in children is limited. Therefore, this study sought to examine the association between social determinants of health and HL in children using the parental socioeconomic status, such as educational attainment level, employment status and income level, non-medical determinants of health (rurality, housing, type of toilet, availability of piped drinking water, and exposure to cigarette smoke) as proxy factors for social determinants of health in children. This was a secondary data analysis of a cross-sectional survey conducted with 517 children in South Africa. We conducted multivariable logistic regression to test for the association between HL and exposure variables such as non-medical determinants of health and parental socioeconomic status using Stata v18 for Macintosh. Odds ratios (OR) with 95% confidence intervals (CIs) were used to ascertain the odds of HL with exposure variables. One hundred and two participants (n = 102, 19.7%) had HL, including 57 (55.9%) females. Crude analysis showed increased odds of HL in females (OR:1.6; 95%CI: 1.0 – 2.5, *P = 0.03*) and children younger than9 years (OR: 2.0; 95%CI: 1.3 – 3.1, *P = 0.003*). After adjusting for age and sex, exposure to cigarette smoke (aOR: 4.0; 95%CI:2.4 – 6.4, *P < 0.001*), living in a mud house (aOR: 1.6; 95%CI:1.2 – 2.7, *P = 0.04*), lack of piped drinking water (aOR: 1.9; 95%CI:1.1 – 3.1, *P < 0.02*), using pit latrines (aOR: 4.1; 95%CI:1.3 – 13.0, *P = 0.01*), having parents who (i) did not complete high school (aOR: 2.8; 95%CI:1.4 – 2.4, *P = 0.01*), or those earning a combined annual household income (iii) less than $2,882 (aOR: 6.2; 95%CI:2.1 – 51.1, *P = 0.03*) or (iv) between $2,883 and $8,006 (aOR:5.0; 95%CI:2.5 – 43.5, *P = 0.05*) increased the odds of HL. Based on these findings, we recommend public health interventions targeting these social determinants to reduce the global burden of HL, and further research to understand the pathophysiology of HL in those exposed to smoking or using pit latrines.

## Introduction

Globally, over 34 million children below 15 years of age are living with hearing loss (HL) with over 60% of cases due to preventable causes [1]. Approximately 80% of this HL occurs in low- and middle-income countries, including Sub-Saharan Africa [1]. In South Africa (SA), the prevalence of childhood HL ranges from 2.2% to over 32% [2] among school-aged children (7–17old) [3]. Unfortunately, this variability in the literature on HL in SA may undermine the importance of public health policies and interventions for ensuring good paediatric ear and hearing health.

In children, HL is either categorised as congenital or acquired, depending on when the it occurred, when it is diagnosed and its aetiology [4]. Congenital HL, which is present at birth, is either genetic or non-genetic, with genetic HL accounting for more than half of congenital HL observed in early childhood [5]. Non-genetic HL is usually caused by maternal exposure to a myriad of conditions grouped as the STORCH Complex, including maternal syphilis, toxoplasmosis, rubella, cytomegalovirus, herpes and other infections including HIV [5]. Besides the STORCH complex, other known aetiologies of congenital HL include congenital anomalies such as cochleovestibular malformations and cochlear nerve deficiency, maternal trauma, the use of ototoxic medications during pregnancy, and other perinatal risk factors such as prematurity, low birth weight, and hyperbilirubinemia [6].

Acquired HL, which can occur at any time during early and late childhood, can result from conditions such as otitis media (OM), cerumen impaction, infections such as HIV, bacterial meningitis, mumps and measles, treatment with ototoxic drugs, and trauma [2,7]. In addition, living conditions have also been linked with HL. For instance, children exposed to cigarette smoke in the household in a study in Kuwait were three times likely to acquire HL compared to those unexposed [8]. Exposure to cigarette smoke is associated with an elevated risk of developing otitis media (OM) [8–10]. This association may be attributable to a compromised immune system and impaired respiratory function, which can result in Eustachian tube dysfunction and transient HL [10–12]. Furthermore, exposure to cigarette smoke is known to adversely affect the cochlear basilar membrane and induce microvascular damage, ultimately leading to the apoptosis of cochlear hair cells and sensorineural hearing loss (SNHL) [13,14].

Unfortunately, childhood HL can leave lifelong consequences for education, health, and well-being [15,16]. It is linked to poor communication development which translates to poor scholastic performance and academic underachievement [17,18]. Additionally, it is associated with increased risks of bullying [19,20], behavioural problems [18], and social isolation [19,21]. Collectively, these factors may translate to poor vocational outcomes and decreased economic activity later in life [22–24].

A growing body of evidence has shown strong associations between disability in general and factors such as poverty, living in marginalised environments, limited access to education, lower levels of educational attainment, unemployment and limited access to healthcare throughout the lifecourse [25,26]. These multifaceted determinants of health, collectively referred to as social determinants of health, are linked to an increased vulnerability to disability, which emphasises the importance of addressing these systemic disparities to promote better health outcomes. Data from high income countries show that children with HL are poorer [27], leading to worse educational outcomes and reduced access to healthcare [28]. In addition to poverty, poor living conditions have also been correlated with increased risk of HL [9,14,29]. Thus, it is important to identify the determinants associated with HL in children to inform public health programmes that aim to reduce its global burden. Thus, this study examines the association between social determinants of health and HL in children in SA.

## Materials and methods

This was a secondary data analysis of data from a cross-sectional study.

### Study population

We used secondary data from a cross-sectional study conducted in Mthatha, SA, between 15 July and 31 December 2022. The primary study included individual-level data of 517 participants and included covariate data obtained from hearing screenings and individual case histories for each participant. As per the primary study, children aged 5–12 years underwent a hearing screening if they could be conditioned for testing. Exclusion criteria in the primary study included babies younger than 5 years as they are difficult to condition and requires experience in assessing paediatric patients while those who were older than 12 years are not deemed paediatric patients at the data collection site. Additionally, those with complex conditions such cerebral palsy and those with difficulty following instructions were also excluded. Primary caregivers of the participants in the parent study provided written consent for their minor children to participate in the study and for their data to be used for research purposes, including secondary data analysis. Children who were old enough to understand instructions give assent provided verbal assent to participate in the primary study.

### Study context

Mthatha is a rural town located in the Oliver Reginald Tambo District, with an estimated population of 1,514,306 people [30]. In 2019, 53% of the population was females with a median age of 19 years while over a quarter (27%) of the population was children under 14 years old, indicating a need for service provision and social development initiatives aimed at youth [30]. The population is 99% African, and an estimated 66.5% (n = 1 006 431) of the total population lived in poverty, with one of the lowest per income capita in SA [30].

Twenty-seven percent (27%) of households in the Oliver Reginald Tambo district, where Mthatha is located, are unable to afford enough food to sustain themselves for an entire month due to poverty, 683,584 people are grant dependents in this area [30]. Only 10.9% (n = 37 900) of the households have piped water inside the dwelling, and 61.9% of households do not have access to piped water [30].

According to the Department of Cooperative Governance and Traditional Affairs, over 70% of the population have not completed high school education, i.e., 10% have completed Grade 9 while the remaining 60% have only completed grades 10–11 [30]. In addition, only 22% of the population have a matric certificate, including less than 10% with higher education (graduate degrees and diplomas). Of the total population, only 15% (n = 221 000) were employed in 2019 and approximately 26% (n = 400,941) of those employed earned between $5.5 to $21.86 (USD) per month, which accounts for the highest number of earners, followed by income levels of $43.78 to $87.45 per month. Over a third (39.6%, n = 599 829) of the population receive no income at all, relying on social grants. The average combined annual household income was $798.01 in 2019, significantly lower than the national average of $16,596.79 [30]. Hearing healthcare in the Eastern Cape province, where this study was conducted is grossly unavailable, with only 29 of the 92 state hospitals and one primary healthcare clinic offering audiology services [31].

### Hearing test

All participants underwent an otoscopic examination using the Mini Heine 3000 fibreoptic otoscope to identify any outer or middle ear pathology that could lead to HL such as impacted wax and OM. All participants with impacted cerumen underwent its removal prior to hearing assessments. After an otoscopic examination, all participants underwent pure tone audiometric screening using the hearScreen^TM^ to detect the presence of HL at 20 dB across 500 – 4000 Hz in each ear. All hearing screenings were conducted in a quiet room. The hearScreen^TM^ is a portable pure tone audiometric screening test calibrated according to international standards and continuously monitored ambient noise during screening. If any noise exceeded the limit set by international standards, the hearing screening test was paused until ambient noises decreased to minimum acceptable levels [32]. All participants who failed the hearing screening were referred for further diagnostic evaluation at a local Hospital. To pass the hearing screening, each participant had to signal that they heard a pure tone at all four test frequencies at 20dB. This was chosen as normal hearing in children is typically described as hearing sensitivity at 15dB and below [33].

### Variables

#### Hearing loss.

HL was defined as thresholds greater than15 dB at any test frequency (250 – 8000 Hz) in either of the two ears [33]. HL can also be classified into conductive, sensorineural, or mixed. Conductive HL (CHL) occurs when sound transmission is obstructed in the outer or middle ear, often due to blockages such as impacted cerumen or OM. In contrast, SNHL results from damage to the inner ear or auditory nerve, typically leading to a more permanent HL. Mixed hearing loss comprises both conductive and sensorineural elements, affecting the outer and middle ear as well as the inner ear [7]. The following normative values are used to grade the severity of HL in children: (i) normal hearing 15 dB HL or less, slight from 16 to 25 dB HL, mild from 26 to 40 dB HL, moderate between 41 and 55 dB HL, moderately-severe between 56–70 dB HL, severe between 71–90 dB HL, and profound greater than 91 dB HL [33]. In this study, HL of any type and severity was included and participant hearing outcomes were dichotomised into normal hearing or HL.

#### Indicators of parent socioeconomic status.

Indicators of parent socioeconomic status included highest level of education, employment and combined annual household income. The highest level of education was categorised into (i) graduate education, (ii) Matric Certificate, (iii) incomplete high school education and (iv) primary school or no education. Parents or primary caregivers were also asked to indicate whether they were employed or not, and to approximate their combined annual household income. The self-reported employment status was dichotomised into employed or not, while the combined annual household income was categorised into three categories in line with income status in SA, i.e., (i) $0 – $2882, classified as Poor, (ii) $2,883 – $8,006, classified as Low Emerging Middle Class, and (iii) $ 8,007– $19,214, classified as Emerging Middle Class [34].

#### Non-medical determinants of health.

Non-medical determinants of health included exposure to cigarette smoking in the household, location (i.e., urban, semi-urban and rural), type of dwelling (i.e., cement or mud house), availability of piped drinking water and type of ablution (flushing system or pit latrines). In SA, rural areas are geographical areas located outside towns and cities with low population densities and small settlements while urban areas are densely populated with built-up infrastructure and high population density. Semi-rural areas are transitional zones between rural and urban areas with limited facilities and infrastructure.

#### Covariates.

Age and sex were assessed as covariates in the association between social determinants of health and HL. Age was dichotomised into 5 – 8 and 9 – 12 years old, to correspond with the primary school education phases, with 5–8-years-old important for literacy development. Sex was also dichotomised into male or female.

### Data management and analysis

The secondary data, which was anonymised, i.e., all personal and identifying information removed, was received from the lead investigator of the primary research on 15 July 2023 in a Microsoft Excel spreadsheet. All statistical analysis was performed using Stata v18 [35]. HL was set as the dependent variable while all the other variables were exposure variables. Descriptive statistics in the form of percentages and frequencies were performed to describe categorical data, including hearing status, sex, living location, type of dwelling, availability of piped drinking water, type of toilet, exposure to cigarette smoking, parents’ education level and employment status.

We conducted multivariable logistic regression informed by previous literature to examine the association between social determinants of health and HL. In our model, we incorporated age and sex as potential confounders. Each exposure variable was individually analysed to ascertain crude odds ratios (ORs) and corresponding 95% confidence intervals (CIs), offering insight into the odds of HL in participants exposed to various social determinants of health. An adjusted logistic regression model was the executed, adjusting for age and sex, to obtain adjusted ORs (aOR) and respective 95% CIs. A two-tailed Students t-test was then calculated with significance level set at ≤0.05, to quantify the significance of the difference is between the means of the groups.

### Ethical statements

This study received ethics approval from the Biomedical Research Ethics Committee of the University of KwaZulu-Natal (Ref: BREC/00003877/2022).

All the procedures were performed under the 2013 Helsinki declaration and its later amendments or comparable ethical standards. The primary caregivers of all children in this study gave written consent for their children to participate in this study and all participants verbal assent to participate in the study and for the data to be published in scientific journals. The purpose and the procedures of the study were described, and participants were informed that their participation was voluntary, they could withdraw at any time or refuse any part of the study and that all collected information would be kept confidential for scientific purposes only.

## Results

### Participant demographic characteristics

Five hundred and seventeen children with a mean age of 8.4 years (SD **± **2.3) participated in this study, of which 53.4% (n = 276) were male. Overall, 19.7% (n = 102) of the participants had HL and female participants accounted for 55.8% (n = 57) of participants with HL.

There were 277 children below the age of eight (8) years and of these, 24.5% (n = 68) had some form of HL, compared to 14.2% (n = 34) of the children aged 9 years and above. In addition, 199 children were exposed to cigarette smoking in the household and of these, 32% (n = 64) had some form of HL. Furthermore, only 199 children had access to piped drinking water, and of these 27.1% (n = 54) presented with HL. Specific to location of the household, those staying in rural areas had the highest prevalence of HL (21.1%, n = 91/431) followed by those living in semi-urban areas (13%, n = 9/69). Similarly, there was a higher prevalence of HL in children living in mud houses (27.5%, n = 53/193) when compared to those living in concrete dwellings (15.1%, n = 49/324). children with flushing toilets had a lower prevalence of HL (6%, n = 6/100) when compared to those using pit latrines (23%, n = 96/417). Table 1 below shows the distribution of participant characteristics (n = 517) stratified by hearing status.

**Table 1:** Participant Demographic Characteristics.

Variable	Normal, n(%) n = 415	HL, n(%) n = 102	Total, n(%) n = 517
**Participant Characteristics**			
***Age***			
9 – 12 years old	206 (85.8%)	34 (14.2%)	240 (46.4%)
**5** – **8-years-old**	209 (75.5%)	68 (24.5%)	277 (53.6%)
***Sex***			
**Male**	231 (83.7%)	45 (16.3%)	276 (53.4%)
**Female**	184 (76.34%)	57 (23.7%)	241 (46.6%)
**Household Characteristics**			
***Cigarette Smoke Exposure***			
**No**	280 (88%)	38 (12%)	318 (61.4%)
**Yes**	135 (67.8%)	64 (32.2%)	199 (38.6%)
***Home Location***			
**Urban**	15 (88.2%)	2 (11.8%)	17 (3.2%)
**Semi-Urban**	60 (87%)	9 (13%)	69 (13.4%)
**Rural**	340 (78.9%)	91 (21.9%)	431 (83.4%)
***Type of Dwelling***			
**Concrete House**	275 (84.9%)	49 (15.1%)	324 (62.7%)
**Mud-House**	140 (72.5%)	53 (27.5%)	193 (37.3%)
***Has Piped Drinking Water***			
**Yes**	269 (84.9%)	49 (15.1%)	317 (61.3%)
**No**	145 (72.9%)	53 (27.1.%)	199 (38.7%)
***Type of Ablution Facilities***			
**Flushing system**	94 (94%)	6 (6%)	100 (19.3%)
**Pit latrine**	321 (77%)	96 (23%)	417 (80.7%)

### Parental socioeconomic status

The highest prevalence (22.6%, n = 46/203) of HL was observed in in children whose parents had not completed high school education, followed by those whose parents had completed matric education (20.6%, n = 41/199). In addition, 22% (n = 70/318) of children whose parents were unemployed also presented with HL. In terms of combined annual household income, children from the lowest household income had the highest prevalence of HL, with 22.5% (n = 87/387) of these children presenting with some form of HL. Table 2 below summarises the parents’ socioeconomic indicators for each child that participated in this study.

**Table 2:** Parental Socioeconomic Status.

Variable	Normal, n(%) n = 415	HL, n(%) n = 102	Total, n(%) n = 517
**Parent socioeconomic status**			
***Highest education level***			
Graduate	100 (87%)	*15 (13%)*	115 (22.2%)
Matriculation	158 (79.4%)	*41 (20.62%)*	199 (38.5%)
Did not complete high school	157 (77.3%)	*46 (22.6%)*	203 (39.3%)
***Employment status***			
Employed	167 (83.9%)	*32 (16.1%)*	199 (38.5%)
Unemployed	248 (78%)	*70 (22%)*	318 (61.5%)
***Combined Annual Income***			
$0 – $2882	300 (77.5%)	87 (22.5%)	387 (74.9%)
$2,883 – $8,006	72 (83.7%)	14 (16.8%)	86 (16.6%)
$8,007+	43 (97.7%)	1 (2.3%)	44 (8.5%)

### Social determinants of health associated with hearing loss

Crude analyses found that in this population, the odds of experiencing HL were higher if children were female (OR:1.6; 95%CI: 1.0 – 2.5, *P = 0.03*) and younger than 9 years old (OR: 2.0; 95%CI: 1.3 – 3.1, *P = 0.003*), exposed to cigarette smoking (OR: 3.5; 95%CI: 2.2 – 5.5, *P < 0.001*), lived in a mud house (OR:2.1; 95%CI:1.4 – 3.3, P = 0.001), lacked access to piped drinking water (OR: 2.1; 95%CI: 1.4 – 3.2, *P = 0.001*), used pit latrine for ablution (OR: 4.7; 95%CI: 2.0 – 11.0, *P < 0.001*), and had parents who did not complete high school (OR:2.0; 95%CI: 1.1 – 3.7, *P = 0.03*) (Table 3). In addition, having parents with combined annual household income less than $2,882 (OR: 12.5; 95%CI: 1.7 – 92.0, *P = 0.01*) or combined annual household income between $2,883 and $8,006 (OR: 8.4; 95%CI: 1.1 – 65.8, *P = 0.04*) increased the odds of HL in this population. However, the confidence intervals for both these associations were large, due to a smaller sample size. There was no evidence for an association between home location (rurality) (OR: 2.0; 95%CI: 0.5 – 8.9, *P = 0.36*) or being unemployed (OR: 1.5; 95%CI:0.9 – 2.3, *P = 0.10*) and HL.

**Table 3:** Logistic regression analysis of hearing loss and social determinants of health.

Variable	*n* with HL (%)	OR (95% CI)	*P-value*	Adjusted OR (95% CI) †	*P-value*
Sex					
* Male*	*n = 45/276 (16%)*	*Reference*			
* Female*	*n = 57/241 (23.7%)*	1.6 (1.1 – 2.5)	P = 0.03*		
Age					
* 9 – 12 years*	*n = 34/240 (14.2%)*	*Reference*			
* 5 – 8 years*	*n = 68/277 (24.6%)*	2.0 (1.3 – 3.1)	P = 0.003*		
Exposure to Cigarette Smoking					
* No*	*n = 38/318 (11.9%)*	*Reference*		*Reference*	
* Yes*	*n = 64/199 (32.2%)*	3.5 (2.2 – 5.5)	*P < 0.0001*	*4.0 (2.4 – 6.4)*	P < 0.001*
Home Location					
* Urban*	*n = 2/17 (11.8%)*	*Reference*		*Reference*	
* Semi-urban*	*n = 9/69 (13%)*	1.1 (0.2 – 5.8)	*P = 0.89*	0.9 (0.1 – 5.7)	*P = 0.87*
* Rural*	*n = 91/431 (21%)*	2.0 (0.45 – 8.9)	*P = 0.36*	0.4 (0.1 – 2.2)	*P = 0.26*
Type of Dwelling					
* Cement House*	*n = 49/324 (12%)*	*Reference*		*Reference*	
* Mud House*	*n = 53/193 (27.5%)*	2.1 (1.4 – 3.3)	*P = 0.001*	1.6 (1.2 – 2.7)	P = 0.04*
Availability of Piped Drinking Water					
* Yes*	*n = 48/318 (15.1%)*	*Reference*		*Reference*	
* No*	*n = 54/199 (27%)*	2.1 (1.5 – 3.2)	*P = 0.001*	1.9 (1.1 -3.1)	P = 0.02*
Type of Ablution Facilities					
* Flushing Toilet*	*n = 6/100 (6%)*	*Reference*		*Reference*	
* Pit Latrine*	*n = 96/417 (23%)*	4.7 (2.0 – 11.0)	*P < 0.0001*	4.1 (1.3 – 13.0)	P = 0.01*
Parent’s Education Level					
* Graduate degree*	*n = 15/115 (13%)*	*Reference*		*Reference*	
* Matric Certificate (GCSE ABC Equivalent)*	*n = 41/199 (20.6%)*	1.7 (0.9 – 3.3)	*P = 0.09*	0.9 (0.6 – 1.7)	*P = 0.72*
* Did not complete High School Education*	*n = 46/203 (22.7%)*	2.0 (1.1 – 3.7)	*P = 0.03*	2.8(1.4 – 2.8)	P = 0.01*
Parents’ Employment Status					
* Employed*	*n = 32/199 (16%)*	*Reference*		*Reference*	
* *Unemployed	*n = 70/318 (22%)*	1.5 (0.9 – 2.3)	*P = 0.10*	1.6 (1.1 – 2.6)	P = 0.05*
Parents’ Combined Household Income in USD					
* $8,007+*	*n = 1/44 (2%)*	*Reference*		*Reference*	
* $2,883 – $8,006*	*n = 14/86 (16.3%)*	8.4 (1.1 – 65.8)	*P = 0.04*	5.0 (2.4 – 43.5)	P = 0.05*
* $0 – $2882*	*n = 87/387 (22.5%)*	12.5 (1.7 – 92.0)	*P = 0.01*	6.2 (2.1 – 51.1)	P = 0.03*

Note: HL = hearing loss, OR=Odds Ratio, CI = Confidence Interval, USD = United States Dollar. † = Adjusted for Sex and Age, * = Statistically Significant.

Adjusted effect estimates when accounting for participant age and sex showed that exposure to cigarette smoking (aOR: 4.0; 95%CI:2.4 – 6.4, P < 0.001), living in a mud house (aOR: 1.6; 95%CI:1.2 – 2.7, *P = 0.04*), lack of piped drinking water (aOR: 1.9; 95%CI:1.1 – 3.1, *P < 0.02*), using pit latrines (aOR: 4.1; 95%CI:1.3 – 13.0, *P = 0.01*), having parents who did not complete high school (aOR: 2.8; 95%CI:1.4 – 2.4, *P = 0.01*), parental unemployment (aOR: 1.6; 95%CI:1.1– 2.6, *P = 0.05*), a combined annual household income less than $2,882 (aOR: 6.2; 95%CI:2.1 – 51.1, *P = 0.03*) or those earning between $2,883 and $8,006 annually (aOR:5.0; 95%CI:2.5 – 43.5, *P = 0.05*) were all associated with a higher odds of HL.

## Discussion

This is the first study to examine the association between social determinants of health and HL in children in SA and findings from this study contribute to the growing body of evidence globally that examines these associations. In this study, females and younger participants were more likely to have HL when compared to male and older participants. In addition, exposure to cigarette smoke, living in a mud house, using pit latrines, and lacking access to piped drinking water were all associated with higher odds of HL.

The factors associated with HL in our study align with some of the emerging evidence globally. For instance, the contextual factor with the highest odds of HL in this study was the use of pit latrines, even when accounting for participant age and sex, and exposure to cigarette smoking within the household. While a clear link between the proper disposal of excreta and improved health has been established [36], there is limited research examining the association between the use of pit latrines and HL. Nevertheless, two South African studies in KwaZulu-Natal identified high concentrations of *Staphylococcus aureus* [37] and *Escherichia Coli* (*E. Coli*) [38] on the surface areas of pit latrines between 2017 and 2018. *E. Coli* and *Staphylococcus aureus* are common pathogens that cause OM in children, as these pathogens are often introduced into the ear canal and subsequently the middle ear cavity through contact with contaminated surfaces. This leads to the development of OM [39], which is usually accompanied by conductive HL. Thus, it is plausible that using pit latrines increases the risk of contracting *E. Coli* and *Staphylococcus* aureus, leading to OM and HL.

Tiwari et al [40] argues that the decision regarding the choice of ablution facilities is complex and can be affected by other factors such as the socioeconomic status and the availability of piped water for flushing. This suggests that a connection between the use of pit latrines, availability of piped water and socioeconomic status exists. In this study, children without access to piped drinking water were twice as likely to have HL when compared to those with access to piped drinking water, and this association remained significant even after adjusting for smoking exposure, age, and sex. As a result, it is difficult to determine if the increased odds of HL in children using pit latrines was due to conductive HL, lower socioeconomic status, unavailability of water in the household or a combination of all these factors.

We also found over four times the odds of HL in children exposed to smoking in the households when compared to children who were not exposed to smoking, which has been demonstrated in previous studies [8,9,14,29]. While the exact pathophysiology remains unclear, there is an increased risk of OM in children exposed to smoking due to the suppression of the immune system, enhancement of bacterial adherence factors, the exposure to toxins, and impairment of the respiratory muco-ciliary apparatus. This often leads to Eustachian tube dysfunction and the resulting OM and HL [11,12]. More than 60% of those exposed to smoking a study by Csákányi *et al* [11] study also had OM, linking smoking exposure to OM and conductive HL. In other studies, smoking exposure have led to SNHL, which is associated with the dysfunction of endothelial physiology of the cochlea, microvascular impairment, and apoptosis of the cochlear hair cells [13,14]. Since the cochlea relies on sufficient blood supply, it is vulnerable to changes in blood flow, which can lead to SNHL [41].

Our findings also showed higher odds of HL in children whose parents had lower income levels, which is similar to a previously reported study. For instance, The Third National Health and Nutrition Examination Survey among 6 166 children aged 6 – 19 years of age from the United States of America showed a higher prevalence (16.3%) of HL amongst children from low-income families when compared to children from high-income families (7.9%) [42]. This could be due to the fact that children from low-income families may face barriers to accessing timely and effective hearing healthcare such as diagnostic testing and may also experience delays or even lack access to necessary medical care for conditions such as OM that are known to cause HL [43]. We also found significant associations between lower parental education level and increased odds of HL. While there is no clear link between parental education level and HL, our findings could be due to the fact that parents who do not complete high school education are more likely to be unemployed, leading poverty. A recent study in South Africa found that those who do not complete high school education have, on average, an 82% probability of being poor and living in poverty [44]. Thus, children whose parents have lower educational attainment, i.e., those who did not complete high school, are more likely to face the same effects as children whose parents have lower income levels.

Findings from our study also showed increased odds of HL in those living in rural areas. However, the 95% confidence interval of this estimate crossed 1 (95% CI: 0.45 – 8.9) with a large p-value, i.e., p = 0.82, which indicates that this result could be due to chance. Nevertheless, this high OR could be due to the fact that HL is positively correlated with other indicators for a lower socioeconomic status such as poverty, lower income class, unemployment, and poor living conditions, which are more prevalent in rural settings and were found to be significantly associated with HL in our present study [45].

Our study has strengths and limitations to consider. The careful calculation of sample size, with high statistical power and representativeness of the target population, minimises random error and selection bias. The comprehensive dataset without missing data ensures the accuracy of the analysis. The data collected by an experienced Audiologist and double-checked data entries contribute to the study’s reliability. Furthermore, the inclusion of participants from diverse socioeconomic areas and locations within Mthatha enhances the potential generalisability of the findings to other areas with similar demographics. However, limitations include the cross-sectional design, which hinders the establishment of causal relationships between variables. Some information reported on the socioeconomic status may not be accurate as participants may have given false information for social desirability. Furthermore, unmeasured variables and the absence of classification of HL types and severity limit the study’s ability to explore the impact of specific factors on HL types.

### Based on the findings from this study, the following recommendations were made:

#### Recommendations for public health policy and practice.

Public health interventions aimed at minimising household exposure to cigarette smoking, increasing access to piped and safe drinking water, increasing access to safe housing and improved educational attainment and employment are recommended to ensure a more confident and self-sufficient society. In addition, we recommend interventions to raise awareness and increase hygiene practices around pit latrine users, such as improving handwashing practices, and ensuring that the pit latrines are regularly cleaned and disinfected. As such, policymakers, public health authorities and multi-ministry collaboration is required to increase awareness, re-evaluate and ensure enforcement of Tobacco Control guidelines. Additionally, these collaborative efforts should ensure the development and implementation of policies that improve access to piped drinking water, improve sanitation and hygiene practices for those who use pit latrines to ensure that children are better protected against such exposure. Furthermore, there is a need to widen participation and increase educational attainment, which could be attained through Adult Based Education or Technical and Vocational Education and Training, and improved access to educational funding for those who want to further their education through bursaries and the National Student Financial Aid. Moreover, periodic screening to ensure timely detection and the provision of targeted interventions such as the fitting of hearing aids and provision of aural rehabilitation are recommended to mitigate the impact of HL observed in the participants in this study.

#### Recommendations for future research.

Further longitudinal research is recommended to better establish causal relationships between the social determinants of health identified in this study and HL. Additionally, studies with detailed classification of HL types and severity are recommended to better understand how specific social determinants of health factors affect different forms of HL. We further recommend research to understand the pathophysiology of HL in children exposed to cigarette smoke and those using pit latrines, given the high number of children exposed to cigarette smoke (38%, n = 199) and using pit latrines (80%, n = 417) in the present study. Since this this study was conducted in one province in SA, and that data collection on different populations from other parts of the country may show different findings, further large-scale research is recommended to obtain country-wise estimates of the impact social determinants of health on hearing outcomes in SA.

## Conclusion

These findings contribute to the growing body of research on the relationship between social determinants of health and HL, and suggest that exposure to cigarette smoke, poor living conditions (use of pit latrines, lack of clean piped drinking water and mud housing) and lower socioeconomic status (low educational attainment, parental unemployment, and low household income) may increase the risk of HL in children in SA. These findings are useful in the planning of targeted interventions aimed at reducing the global burden of HL

## Supporting information

S1 Data Raw study data. (XLSX)
